# Hacking Cough

**Published:** 1878-05

**Authors:** 


### HALL'S JOURNAL OF HEALTH.

**Our Legitimate Scope is almost boundless: for whatever begets pleasurable and harmless feelings, promotes Health; and whatever induces disagreeable sensations, engenders Disease.**

**WE AIM TO SHOW HOW DISEASE MAY BE AVOIDED, AND THAT IT IS BEST, WHEN SICKNESS COMES, TO TAKE NO MEDICINE WITHOUT CONSULTING A PHYSICIAN.**

**Vol. 25** **MAY, 1878** **[No. 5.**

### HACKING COUGH.

It is not known as generally as it ought to be, that consumption, that disease known as "common consumption of the lungs," called by many a decline," begins with a regular hacking cough in the morning, or on getting up. In fact it is so slight at first, that it scarcely amounts to a cough; it is a mere "hem" or effort to clear the throat of something which seems to excite a little tickling sensation there, generally referred to the spot known as the hollow, at the bottom of the neck in front. The persou does not imagine that it has any thing whatever to do with the lungs; he insists upon it that it is only in the throat; and for weeks and even months afterwards, when it has become a decided cough, as regular as the rising of the sun, he regards it as an affection of the throat merely, and repeats the saying a thousand times, as if to reassure himself and others of the truth of it; and that if he could only get something to apply to the throat, and take away "that pesky tickling," he would have no cough, and would be as well as he ever was in his life; he strikes upon his chest triumphantly, and exclaims "all right, not the slightest pain or other inconvenience there, anyhow." To remove this tickling he has the fashionable remedy applied; a bit of sponge is dipped into a solution of the nitrate of silver, and passed down to the tickling spot, and lo it is gone! To make the matter more sure, the applications are renewed daily or several times in the course of a week or two; the physician is gratefully and liberally paid and he returns home with a heart so light and buoyant, he is ready to hug and kiss everybody he meets; is quite as jubilant as an honest man, who by great and long continued exertions, has paid the last dollar of indebtedness he owes in the world. In about two years, on an average, the throat swabbed man dies of consumption; because the nitrate of silver only destroys the sensibilities of the part by the greater wounding inflicted, when nature has recovered from the violence offered, the tickling returns, just the same as ever, and then there is the usual routine of syrups, cough drops, lozenges, trochees and the like, all of which contain opium, which like the nitrate of silver, only deadens the sensibilities of the parts, but for a short period.

Consumption is a disease of the lungs themselves, destroying the little air bladders to which reference has been already made. Perhaps a diagram may illustrate more plainly.

